# Progression from Prehypertension to Hypertension and Risk of Gastrointestinal Cancer—A Nationwide Health-Screening Cohort Study

**DOI:** 10.3390/cancers18040594

**Published:** 2026-02-11

**Authors:** Hyejin Yoon, Jiyu Sun, Dong-Woo Choi

**Affiliations:** 1 Cancer Big Data Center, National Cancer Control Institute, National Cancer Center, Goyang-si 10408, Republic of Korea; dbsp4598@naver.com; 2 Integrated Biostatistics Branch, Division of Cancer Data Science, National Cancer Center, Goyang-si 10408, Republic of Korea; jiyu.sun0@gmail.com

**Keywords:** blood pressure trajectory, cancer risk, gastrointestinal cancer, medication adherence, cohort study

## Abstract

**Background/Objectives:** Hypertension is a major risk factor for cardiovascular disease and is associated with gastrointestinal (GI) cancers. However, the association between prehypertension and cancer risk remains uncertain. We investigated the association of progression from prehypertension to hypertension with the risk of GI cancers. **Methods:** We analyzed a Korean health-screening cohort with prehypertension. Blood pressure (BP) at two consecutive screenings defined three groups: reversion to normotension, persistence of prehypertension, and progression to hypertension. Cause-specific Cox proportional hazards models were used to estimate adjusted hazard ratios (aHRs) and 95% confidence intervals (CIs). Sensitivity analyses were conducted by exposure-window (2, 4, and 6 years), lag times (1–5 years), and BP reclassification, along with subgroup and medication adherence (medication possession ratio [MPR]) analyses. **Results:** Of 131,167 individuals, 40,859 (31.2%) reverted to normotension, 62,600 (47.7%) remained prehypertensive, and 27,708 (21.1%) progressed to hypertension. Progression to hypertension was associated with higher overall GI cancer risk (aHR 1.16, 95% CI 1.08–1.26), whereas persistence was not. Site-specific analyses showed stronger associations for colorectal and pancreatic cancers. Among those who progressed, associations were stronger in participants younger than 65 years and in those without diabetes. Excess risk was more pronounced with low adherence (MPR < 0.80) or non-prescriptions. Moreover, sensitivity analyses using the European Society of Cardiology 2024 criteria showed similar results. **Conclusions:** Preventing progression from prehypertension to hypertension may reduce long-term GI cancer burden. Early detection and treatment of new-onset hypertension, with high medication adherence, could help reduce the future burden of GI cancers.

## Simple Summary

Hypertension is a major risk factor for cardiovascular disease and has been linked to gastrointestinal cancers; however, the cancer risk associated with prehypertension remains uncertain. Using a nationwide Korean health-screening cohort of adults with prehypertension, we evaluated gastrointestinal cancer risk according to blood pressure trajectories: reversion to normotension, persistence of prehypertension, and progression to hypertension. During long-term follow-up, progression to hypertension was associated with a higher risk of gastrointestinal cancers overall, with stronger associations for colorectal and pancreatic cancers. Persistence of prehypertension was not associated with overall risk, but increased colorectal and pancreatic cancer risks were observed. Among those who progressed, excess risk was more pronounced in participants with poor adherence to antihypertensive therapy or without antihypertensive prescriptions. These findings suggest that preventing progression to hypertension and improving early treatment adherence may help reduce the future burden of gastrointestinal cancers.

## 1. Introduction

Hypertension is one of the most prevalent chronic diseases worldwide and is a leading cause of cardiovascular disease and premature death [1]. Previous studies have reported significant associations between hypertension and cancers, including those of the genitourinary and gastrointestinal (GI) systems [2,3,4]. GI cancer, which includes cancers of the esophagus, stomach, colorectal, liver, gallbladder, and pancreas, accounts for approximately one-quarter of global cancer incidence and one-third of cancer-related deaths [5]. In South Korea, there were 288,613 newly diagnosed cancer cases in 2023, of which GI cancers accounted for 97,147 cases (33.7%) [6]. In 2024, among 88,933 cancer deaths, GI cancers constituted a substantial proportion of cancer mortality, including liver cancer (11.7%), colorectal cancer (10.9%), pancreatic cancer (9.2%), stomach cancer (8.1%), and gallbladder and other biliary tract cancers (6.3%) [7]. Biologic pathways such as chronic vascular damage, oxidative stress, and the renin–angiotensin system may link elevated blood pressure (BP) to cancer development [8,9,10]. However, it is still unclear whether earlier, borderline elevations such as prehypertension or elevated BP may also be associated with increased cancer risk.

The seventh report of the Joint National Committee (JNC 7) introduced the concept of prehypertension (systolic BP [SBP]: 120–139, diastolic BP [DBP]: 80–89 mmHg), emphasizing the importance of hypertension prevention and early treatment [11]. Individuals with prehypertension have more advanced cardiovascular structural changes compared to those with normal BP [12] and are at a higher risk of developing hypertension, rendering early prevention a necessity. For example, 28.5% progressed within two years in a Chinese study [13] and 17–37% within four years in the Framingham Heart Study, with older age accelerating progression [14].

Despite recognition of cardiovascular risk, evidence linking prehypertension to GI cancer remains limited. The progression from prehypertension to hypertension represents a critical period when vascular damage and systemic inflammation may exacerbate cancer onset, necessitating research that considers this crucial stage. To capture this borderline range as a contiguous construct amenable to transition modeling, we prespecified JNC 7 prehypertension as the baseline inclusion criterion. Because hypertension classification differs across contemporary guidelines, we also prespecified sensitivity analyses with reclassification according to the current American College of Cardiology (ACC), American Heart Association (AHA), and European Society of Cardiology (ESC) thresholds [15,16].

We examined whether progression from prehypertension to hypertension is associated with an increased risk of GI cancers in a nationwide health-screening cohort and evaluated whether antihypertensive medication adherence modifies this risk among individuals with hypertension.

## 2. Materials and Methods

### 2.1. Data Source

This study used the Korean National Health Insurance Service–Health Screening Cohort (NHIS-HEALS) created by the NHIS of Korea. The NHIS is the single insurer in South Korea, covering over 99% of the population. The cohort was developed to enable comprehensive analysis of healthcare utilization and health outcomes among health-screening participants. The database is composed of a 10% random sample (*n* = 514,866) of adults aged 40–79 years who received a general health examination among some 5.15 million eligible individuals. It includes data on eligibility, healthcare utilization, health-screening results, and provider information from 2002 to 2019 [17].

### 2.2. Study Design

We conducted a retrospective cohort study using the NHIS-HEALS data to investigate the risk of GI cancer according to BP trajectories from baseline prehypertension (t0). Participants were aged ≥ 40 years who underwent health screenings between 2003 and 2006 and met the criteria for prehypertension at the first screening (details in Section 2.3.1). To minimize immortal time bias, we used a landmark approach. The index date was defined as one year after the last health screening conducted within four years following the initial screening (t1), and follow-up continued until 31 December 2019, which was the latest available observation point provided by the NHIS at the time of data access (Supplementary Figure S1).

The following exclusion criteria were applied. First, individuals who had not received any health screening within four years after the initial screening were excluded (*n* = 42,007). Second, we excluded subjects with either SBP ≥ 140 mmHg or DBP ≥ 90 mmHg during the 2002 wash-out period. Individuals diagnosed with hypertension and prescribed antihypertensive agents for ≥30 days before the first screening were also excluded (*n* = 94,832). Third, participants with a prior cancer diagnosis before the index date were excluded (*n* = 3741). Fourth, those who died before the index date were removed from the cohort (*n* = 424). Finally, individuals with missing covariate data were excluded (*n* = 17,200). As a result, a total of 131,167 participants were included in the final analysis (Supplementary Figure S2).

This study was approved with an exemption from ethics review by the Institutional Review Board of the National Cancer Center (approval No. NCC2025-0086; approved on 19 March 2025), Korea, and was conducted in accordance with the data use agreement with the National Health Insurance Service (NHIS). All data were anonymized and used solely for research purposes in compliance with the National Health Insurance Act.

### 2.3. Study Variables

#### 2.3.1. Exposure Variable

At baseline (t0), prehypertension was defined as SBP 120–139 mmHg and/or DBP 80–89 mmHg without meeting hypertension criteria (SBP ≥ 140 mmHg or DBP ≥ 90 mmHg) or prior antihypertensive treatment (JNC 7). To classify BP trajectories, we identified for each patient the last screening within 4 years after t0 (t1). Using BP at t1 together with prescription information between t0 and t1, each participant was assigned to one of three categories: (1) reversion to normotension (SBP < 120 and DBP < 80 mmHg, with no antihypertensive prescriptions); (2) persistence of prehypertension (SBP 120–139 or DBP 80–89 mmHg, with no antihypertensive prescriptions); and (3) progression to hypertension (SBP ≥ 140 or DBP ≥ 90 mmHg, or a hypertension diagnosis using the International Classification of Disease, 10th Revision [ICD-10; I10–I13, I15] together with ≥30 days of antihypertensive prescriptions) [18].

#### 2.3.2. Medication Possession Ratio

To evaluate the risk of GI cancer according to hypertension management, this study assessed medication adherence among individuals who progressed from prehypertension to hypertension. Adherence was measured using the medication possession ratio (MPR), calculated based on prescription and visit records during the one-year period following the last health screening within four years of the initial screening. MPR, ranging from 0 to 1, reflects the proportion of time a patient possesses medication; higher values indicate better adherence [19]. It is widely used as an indirect indicator of chronic disease management, particularly in hypertension [20]. In this study, an MPR threshold of 0.8 was applied to analyze cancer risk by adherence level.

#### 2.3.3. Outcome Variable

The outcome was the number of days from the index date to hospitalization due to GI cancer. GI cancer events were defined by the date of hospitalization with a relevant cancer code. GI cancer included esophageal (ICD-10: C15), stomach (C16), colorectal (C18–C20), liver (C22), gallbladder and biliary tract (C23–C24), and pancreatic cancer (C25) [21].

#### 2.3.4. Covariates

Covariates were included to adjust for potential confounding effects. These covariates were selected based on previous studies investigating cancer risk factors [22,23,24,25,26,27]. The covariates included age, sex, residential area (metropolitan, urban, rural), insurance premium, disability status (yes, no), family history of hypertension (yes, no), family history of cancer (yes, no), diagnosis of diabetes (yes, no), diagnosis of dyslipidemia (yes, no), Charlson Comorbidity Index (CCI; 0, 1, 2, ≥3), use of insulin or glucose-lowering agents (yes, no), use of lipid-lowering agents (yes, no), body mass index (normal or underweight, overweight, obese), total cholesterol level, alcohol consumption (rarely, 1–4 times/week, 5–7 times/week), physical activity (rarely, 1–4 times/week, 5–7 times/week), smoking status (never, former, current), and year of initial screening (2003–2006).

Socioeconomic status was proxied by the level of insurance premium and classified into deciles (≤5th decile as “low” and 6th–10th deciles as “high”). Body mass index (BMI, kg/m^2^) was categorized as follows: <23 as “normal or underweight,” 23–24.9 as “overweight,” and ≥25 as “obese”. Medication information was identified using active ingredient codes from claims data to classify antihypertensive agents, lipid-lowering agents, insulin, and glucose-lowering agents. For smoking status, individuals who answered “never smoked” were classified as “never”, those who had quit smoking as “former”, and those who currently smoked as “current”.

The covariate assessment periods varied depending on the nature of each variable. Age, sex, residential area, insurance premium level, disability status, BMI, alcohol consumption, smoking status, and physical activity were assessed based on the year of the last health screening conducted within four years after the initial screening (2003–2006). The Charlson Comorbidity Index, diabetes and dyslipidemia status, and use of antihypertensive and lipid-lowering agents were identified using all medical records between the initial screening and the year of the last screening within the four-year window. Family history was assessed using all available health-screening records from the year of the first screening. If hypertension or cancer was reported at any point, the participant was considered to have a family history.

### 2.4. Statistical Analysis

Baseline characteristics were compared between hypertension progression status groups using one-way ANOVA and chi-square tests. The incidence of GI cancer was compared using cumulative hazard curves and the log-rank test. Cause-specific Cox proportional hazards models were used to calculate the hazard ratios (HRs) and adjusted HRs (aHRs) for overall GI cancer and site-specific cancer risks by BP trajectory, while adjusting for age, sex, residential area, insurance premium level, disability, BMI, alcohol consumption, smoking, physical activity, CCI, diabetes and dyslipidemia, the use of insulin or glucose-lowering and lipid-lowering agents, and family history of hypertension and cancer. The proportional hazards assumption was assessed using the Schoenfeld global test (*p* = 0.485, Supplementary Figure S3). Death and the development of non-GI cancers were treated as competing events and censored at the date of the event in the cause-specific Cox models. Subgroup analysis with all covariates was performed. Furthermore, we estimated the site-specific GI cancer risk by MPR strata. A landmark approach was applied in all analyses to reduce immortal time bias. Sensitivity analyses were performed to assess the temporal robustness of the results, including landmark analyses using different exposure-window intervals between baseline and follow-up screenings (2-, 4-, and 6-year gaps, Supplementary Table S1) and varying lag times (1–5 years after the index date, Supplementary Table S2). In addition, BP trajectories were reclassified according to ACC/AHA 2017 [15] and ESC 2024 [16] criteria (Supplementary Table S3). Statistical analyses were conducted using SAS Enterprise Guide 8.3 (SAS Institute Inc., Cary, NC, USA) and R version 3.5.1 (R Core Team, Vienna, Austria), with *p* < 0.05 considered statistically significant.

## 3. Results

### 3.1. Baseline Characteristics of the Study Population

Table 1 summarizes the baseline characteristics of participants with prehypertension. Of 131,167 individuals, 40,859 (31.2%) were reversion to normotension, 62,600 (47.7%) were persistence of prehypertension, and 27,708 (21.1%) were progression to hypertension during the 4-year classification interval. The progression to hypertension group was older on average (59.2 years), exhibited a higher prevalence of rural residence (58.3%), and demonstrated lower insurance premium tiers (37.6%). All covariates exhibited statistically significant differences across BP trajectory groups (*p* < 0.001).

### 3.2. Cumulative Hazard of GI Cancers by BP Trajectories in Participants with Prehypertension

**Figure 1:**
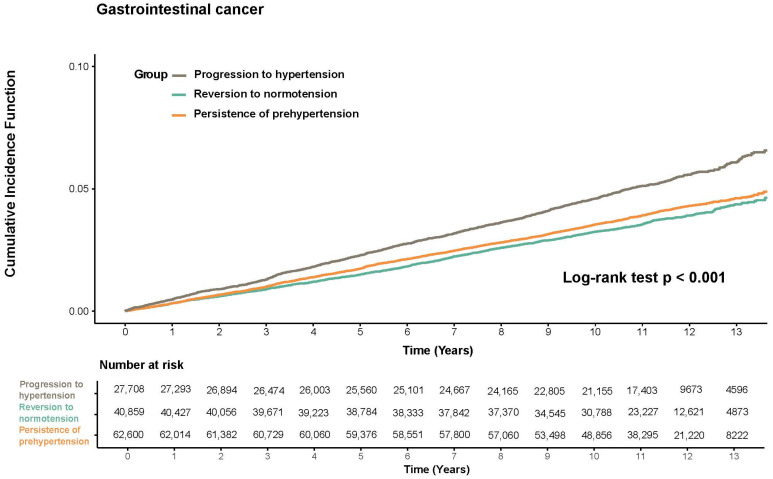
Cumulative hazard of GI cancer by BP trajectories.

Cumulative hazard curves and log-rank tests were used to examine the incidence of GI cancer across BP trajectory groups (Figure 1). The reversion to normotension group maintained the lowest cumulative hazard throughout follow-up, whereas the progression to hypertension group exhibited the greatest increase over time (log-rank test, *p* < 0.001).

### 3.3. Site-Specific Incidence and Hazard Ratios for GI Cancers by BP Trajectory Among Participants with Prehypertension

In Table 2, the progression to hypertension group had a significantly higher risk of overall GI cancer (aHR: 1.16, 95% CI: 1.08–1.26), whereas persistent prehypertension was not associated with risk (aHR: 1.01, 95% CI: 0.95–1.08). Site-specific analyses showed statistically significant associations for colorectal cancer (persistence: aHR 1.14, 95% CI 1.00–1.30; progression: aHR 1.23, 95% CI 1.06–1.43) and pancreatic cancer (persistence: aHR 1.45, 95% CI 1.11–1.91; progression: aHR 1.47, 95% CI 1.07–2.02). There were no significant associations for stomach, liver, and gallbladder and biliary tract cancers; the association with esophageal cancer was marginally significant (aHR: 1.63, 95% CI: 0.98–2.73).

### 3.4. Subgroup Analyses

Subgroup analyses stratified according to age, sex, diabetes, and smoking are shown in Table 3. An age interaction was observed (*p* for interaction = 0.004), and patients aged under 65 in the hypertension progression group exhibited a substantially increased risk of GI cancer compared with the reversion to normotension group (aHR: 1.25, 95% CI: 1.13–1.37). Diabetes status also led to modification of the association (*p* for interaction = 0.044), and the association was stronger among the non-diabetes group (aHR: 1.19, 95% CI: 1.09–1.31) than the diabetes group (aHR: 1.09, 95% CI: 0.94–1.27). There was suggestive evidence of heterogeneity by sex (*p* for interaction = 0.078) and smoking status (*p* for interaction = 0.086). All subgroup analysis results are shown in Supplementary Table S5.

### 3.5. Site-Specific Incidence and Cause-Specific Hazard Ratios for GI Cancers by BP Trajectory and Antihypertensive Adherence Among Participants with Prehypertension

Table 4 shows site-specific GI cancer incidence risks by antihypertensive adherence. Baseline characteristics by antihypertensive adherence (MPR categories) are shown in Supplementary Table S6. The excess risk of GI cancer was pronounced in individuals with low adherence (MPR <0.80; aHR: 1.30, 95% CI: 1.16–1.45) and no prescription (aHR: 1.13, 95% CI: 1.03–1.24). However, participants with high adherence (MPR ≥ 0.80) were not at significantly increased risk of overall GI cancers (aHR: 1.08, 95% CI: 0.95–1.23). When examined by cancer site, risks of esophageal (aHR: 2.24, 95% CI: 1.12–4.47), liver (aHR: 1.52, 95% CI: 1.18–1.95), and pancreatic cancers (aHR: 1.75, 95% CI: 1.14–2.71) were significantly increased with poor adherence. In contrast, elevated risks were also observed in the high-adherence group for esophageal (aHR: 2.32, 95% CI: 1.07–5.03) and pancreatic cancers (aHR: 1.67, 95% CI: 1.03–2.71). For cancers of the stomach or the gallbladder and biliary tract, we observed no statistically significant association.

### 3.6. Sensitivity Analyses

Sensitivity analyses supported the robustness of our findings. Similar results were obtained in the exposure-window analysis, in which varying the interval between baseline and follow-up screenings (2-, 4-, and 6-year gaps) yielded consistent estimates (Supplementary Table S1). As shown in the landmark sensitivity, varying the landmark lag from 1 to 5 years also produced consistent estimates. Persistence of the prehypertension group remained near null across lags. In contrast, progression to the hypertension group showed a stable excess risk at each landmark, with only mild attenuation at longer lags (Supplementary Table S2). Reclassification of BP transitions using ESC 2024 [16] thresholds produced similar effect sizes to the primary analysis. However, when applying ACC/AHA 2017 [15] thresholds, results were attenuated and not statistically significant (Supplementary Table S3).

## 4. Discussion

In a cohort study that followed over 130,000 patients with prehypertension for 10.7 years, progression to hypertension was associated with a 1.16 times increased risk of GI cancer compared to return to normal BP, whereas persistent prehypertension was not. Site-specific analysis revealed increased risks of colorectal and pancreatic cancers, but no significant associations were found for stomach, liver, or biliary tract cancers. Esophageal cancer showed a borderline significant 1.63 times increased risk. Subgroup analyses revealed a stronger association in patients younger than 65 years and without diabetes, and no heterogeneity was observed in other covariates. The results remained consistent across time lags of 1 to 5 years, suggesting minimal immortal time bias and robustness. Among those who developed hypertension, low adherence (MPR < 0.80) and no prescription were associated with a higher risk of GI cancer (1.30 and 1.13 times, respectively), while high adherence (MPR ≥ 0.80) was not. However, the associations by specific cancer site were inconsistent.

These findings support the possibility that the elevation of BP from prehypertension to hypertension could be associated with the onset of GI cancers. A Japanese inpatient cohort study found strong associations between hypertension and several GI cancers, including liver, stomach, colorectal, and pancreatic cancer [28]. Similarly, a large European study showed that higher SBP was associated with overall cancer incidence and mortality. In men, each 10 mmHg increase was linked to higher risks of all cancers, including colorectal, rectal, and pancreatic cancer, while in women, pancreatic and liver cancer were significantly associated [29]. Although previous studies were conducted by different methodologies, their consistent results strengthen the credibility of our findings, though caution is needed given the study’s non-experimental design.

Increasing cancer risk emerged only upon the transition to full hypertension, rather than during stable prehypertensive states, when GI cancers were considered as a composite outcome. However, in site-specific analyses, colorectal and pancreatic cancers showed elevated risks even during the prehypertensive stage, and these risks remained significant after progression to hypertension. This is consistent with previous studies suggesting that increasing BP reflects a long-term metabolic burden and low-grade inflammation that may foster tumor growth [30,31,32,33]. For example, chronic inflammation [10,32], oxidative stress [10], damage to blood vessel linings [8,34,35], and renin–angiotensin system activation [9,36] have been implicated in carcinogenesis. Such mechanisms may be relevant to GI cancers and may be particularly important for colorectal and pancreatic cancers, as suggested by prior mechanistic studies [37,38,39,40,41]. Thus, these findings suggest that individuals with unfavorable BP trajectories—such as progression from prehypertension to hypertension or persistent/worsening prehypertension—may warrant closer clinical attention with respect to GI cancer risk, with potentially more pronounced relevance for colorectal and pancreatic cancers. Further evidence is needed to establish the most appropriate preventive strategies. In clinical practice, BP trajectory information may help identify subgroups with unfavorable BP trajectories, supporting intensified efforts toward earlier and sustained BP control and reinforcing adherence to guideline-based colorectal cancer screening. It may also support heightened clinical vigilance for pancreatic cancer–related symptoms among individuals with these trajectories.

In our cohort, among participants diagnosed with hypertension, those with poor adherence (MPR < 0.80) showed a higher incidence of GI cancers, whereas those with consistent medication use had reduced or neutral associations. Although adherence is not the gold standard for achieving BP control, it may capture patient engagement and broader health behaviors, and it is widely recognized as an important determinant of BP management and related clinical outcomes [42]. On the other hand, participants who met the criteria for hypertension but received no antihypertensive medication had a 13% higher GI cancer risk than those with normotension, consistent with previous studies reporting increased cancer risk in individuals with untreated hypertension or elevated BP states [30,31]. These observations suggest that poor adherence and untreated hypertension may be associated with higher cancer risk, although causal pathways require further investigation. Therefore, early detection of elevated BP and sustained adherence to therapy should be considered not only for cardiovascular protection but also for their potential implications for cancer risk, which warrant further study [43,44]. However, the elevated risks observed in the high-adherence group for certain cancers, such as esophageal and pancreatic cancer, may reflect limited sample size, patient-specific factors, or unmeasured confounders; thus, caution is needed in interpretation. Further research is needed to clarify the effects of antihypertensive drug types, achieved BP control levels, and treatment duration on cancer risk [45,46].

Subgroup analysis revealed age-related differences in GI cancer risk. Individuals under 65 who progressed to hypertension had a significantly higher risk compared to the normotensive recovery group. While younger adults are generally perceived to have a lower absolute cancer risk, cumulative or worsening trajectories of metabolic abnormalities have been associated with increased cancer risk, suggesting that prolonged exposure to metabolic burden may contribute to elevated cancer risk over time [47,48]. These findings could prove the need for proactive BP management in middle-aged populations as part of cancer prevention strategies. In addition, among individuals without diabetes, hypertension progression was associated with an elevated risk of GI cancers, whereas no additional cancer risk was observed among individuals with diabetes, likely due to a saturation of pro-oncogenic metabolic pathways already activated in diabetic states [49]. Diabetes is a severe metabolic dysfunction characterized by hyperglycemia, insulin resistance, and chronic inflammation, all of which are potent carcinogens [50]. In patients with diabetes, these pathways are already activated, creating a high risk of cancer. The addition of other risk factors, such as hypertension, may not significantly increase the already saturated cancer risk [51,52]. These findings were one of the possible explanations that incident hypertension may introduce pro-oncogenic exposures in a relatively healthy metabolic environment; however, causal inference cannot be established.

We conducted sensitivity analyses to assess the robustness of our findings using different definitions of hypertension criteria according to the ESC 2024 [16] and ACC/AHA 2017 [15] guidelines (Supplementary Table S3). Using ESC 2024 [16] thresholds reproduced the primary findings. However, under ACC/AHA 2017 [15], the narrower baseline reduced the sample size and increased the proportion of elderly, leading to wider CI and loss of statistical significance despite similar directions of effect (Supplementary Table S4). Therefore, the lack of significant associations under ACC/AHA guidelines likely reflects these methodological differences rather than contradictory findings. Our results support using a contiguous borderline BP category, such as defined by JNC 7 or ESC 2024 [16], for transition analyses.

This study has several limitations. First, as a claims data-based retrospective study, there are inherent limitations in the ability to control some variables as rigorously as prospective studies, and causal inference cannot be established. Therefore, it should be interpreted with caution. Second, there is the potential for miscoding or the lack of diagnostic codes in the claims data. However, we attempted to increase the robustness of the analyses by adding health-screening data that were linked to claims records to cover covariates as much as possible, and by employing a large nationwide cohort with uniform criteria and long-term follow-up. Third, we could not adjust for multiple potential confounders. Fourth, the results are derived from a Korean screening cohort and may not be applicable to other countries, ethnic groups, or age demographics, where risk-factor profiles and screening practices may differ. It is important to replicate these findings in varied populations for external validation. Finally, we did not investigate site-specific biological mechanisms underlying carcinogenesis. Therefore, further study is needed to clarify these associations in a clinical trial setting.

Nevertheless, there are strengths to our study. First, while previous studies were restricted to only one of hypertension and prehypertension, this study offers an advancement as it elucidates the risk for GI cancer by subsequent blood-pressure change in individuals with prehypertension. This approach is consistent with routine clinical practice. Second, we performed the analysis not only for overall GI cancer but also for site-specific cancers, which may be helpful in personalizing cancer screening by organ in considering future screening guidelines. Persistent prehypertension was linked to significantly higher risks of colorectal and pancreatic cancers, signifying the need for early normalization of BP even in the presence of prehypertension. Third, medication adherence and the related risk of GI cancer were additionally analyzed in the study among patients who developed hypertension.

## 5. Conclusions

Our findings suggest that preventing progression from prehypertension to hypertension could contribute to long-term reductions in the burden of GI cancer, particularly colorectal and pancreatic cancers, and that younger and non-diabetic individuals are likely to benefit more. The increased risk of GI cancer among individuals with low adherence or non-prescribed treatment for hypertension emphasizes the importance of ongoing effective management of hypertension for cancer prevention and highlights the importance of early detection and organized care.

## Figures and Tables

**Table 1:** Baseline characteristics by BP trajectory from prehypertension.

Variables	BP Trajectory from Prehypertension				*p* Value
	Overall	Reversion to Normotension	Persistence of Prehypertension	Progression to Hypertension	
Participants, *n*	131,167	40,859	62,600	27,708	
Follow-up period (years) ^†^	10.7 (2.6)	10.7 (2.5)	10.8 (2.5)	10.7 (2.9)	<0.001
Blood pressure					
Systolic (mmHg) ^†^	124.0 (7.0)	122.4 (6.9)	124.1 (6.9)	125.9 (6.9)	<0.001
Diastolic (mmHg) ^†^	77.7 (5.6)	77.2 (5.7)	77.9 (5.5)	78.3 (5.6)	<0.001
First screening year (years)					<0.001
2003	71,793 (54.7)	19,469 (47.6)	34,442 (55.0)	17,882 (64.5)	
2004	31,161 (23.8)	10,532 (25.8)	15,072 (24.1)	5557 (20.1)	
2005	12,498 (9.5)	4829 (11.8)	5795 (9.3)	1874 (6.8)	
2006	15,715 (12.0)	6029 (14.8)	7291 (11.6)	2395 (8.6)	
Age (years) ^†^	55.52 (8.70)	53.98 (7.85)	54.90 (8.37)	59.18 (9.55)	<0.001
Aged 65 or above	19,970 (15.2)	4141 (10.1)	8168 (13.0)	7661 (27.6)	<0.001
Women	62,403 (47.6)	20,821 (51.0)	27,709 (44.3)	13,873 (50.1)	<0.001
Region					<0.001
Metropolitan	21,041 (16.0)	6719 (16.4)	9851 (15.7)	4471 (16.1)	
Urban	38,195 (29.1)	12,198 (29.9)	18,907 (30.2)	7090 (25.6)	
Rural	71,931 (54.8)	21,942 (53.7)	33,842 (54.1)	16,147 (58.3)	
Household insurance rate					<0.001
Low (≤5th decile)	45,058 (34.4)	13,620 (33.3)	21,027 (33.6)	10,411 (37.6)	
High (6–10th decile)	86,109 (65.6)	27,239 (66.7)	41,573 (66.4)	17,297 (62.4)	
Disability	7867 (6.0)	2100 (5.1)	3552 (5.7)	2215 (8.0)	<0.001
Family history					
Hypertension	36,605 (27.9)	10,103 (24.7)	17,057 (27.2)	9445 (34.1)	<0.001
Cancer	47,424 (36.2)	15,868 (38.8)	22,671 (36.2)	8885 (32.1)	<0.001
CCI					<0.001
0	96,293 (73.4)	29,551 (72.3)	46,991 (75.1)	19,751 (71.3)	
1	20,272 (15.5)	6736 (16.5)	9345 (14.9)	4191 (15.1)	
2	8819 (6.7)	2823 (6.9)	3917 (6.3)	2079 (7.5)	
≥3	5783 (4.4)	1749 (4.3)	2347 (3.7)	1687 (6.1)	
Diabetes	24,946 (19.0)	6293 (15.4)	10,547 (16.8)	8106 (29.3)	<0.001
Dyslipidemia	33,810 (25.8)	9074 (22.2)	14,518 (23.2)	10,218 (36.9)	<0.001
Insulin or glucose–lowering agents	9231 (7.0)	2045 (5.0)	3780 (6.0)	3406 (12.3)	<0.001
Lipid–lowering agents	22,992 (17.5)	6124 (15.0)	9828 (15.7)	7040 (25.4)	<0.001
BMI (kg/m^2^)					<0.001
Normal or underweight	53,084 (40.5)	20,209 (49.5)	24,209 (38.7)	8666 (31.3)	
Overweight	38,125 (29.1)	11,431 (28.0)	18,636 (29.8)	8058 (29.1)	
Obese	39,958 (30.5)	9219 (22.6)	19,755 (31.6)	10,984 (39.6)	
Total cholesterol (mg/dL) ^†^	199.3 (36.5)	196.3 (35.5)	200.1 (36.1)	201.9 (38.4)	<0.001
Alcohol consumption					<0.001
Rarely	101,189 (77.1)	32,065 (78.5)	47,345 (75.6)	21,779 (78.6)	
1–4 times per week	25,426 (19.4)	7740 (18.9)	13,046 (20.8)	4640 (16.7)	
5–7 times per week	4552 (3.5)	1054 (2.6)	2209 (3.5)	1289 (4.7)	
Physical activity					<0.001
Rarely	60,089 (45.8)	17,897 (43.8)	28,111 (44.9)	14,081 (50.8)	
1– 4 times per week	54,896 (41.9)	17,811 (43.6)	26,923 (43.0)	10,162 (36.7)	
5– 7 times per week	16,182 (12.3)	5151 (12.6)	7566 (12.1)	3465 (12.5)	
Smoking status					<0.001
Never	95,474 (72.8)	29,815 (73.0)	44,535 (71.1)	21,124 (76.2)	
Former	12,599 (9.6)	3754 (9.2)	6383 (10.2)	2462 (8.9)	
Current	23,094 (17.6)	7290 (17.8)	11,682 (18.7)	4122 (14.9)	

^†^ Mean and standard deviation. Abbreviations: BP, blood pressure; CCI, Charlson Comorbidity Index; BMI, body mass index.

**Table 2:** Site-specific GI cancer hazard ratios by BP trajectory from prehypertension with cause-specific Cox models.

Cancer Site	BP Trajectory from Prehypertension	Cases/Participants, *n* (%)	Incidence Rate per 100,000 PY	HR (95% CI)	*p* Value	aHR (95% CI)	*p* Value
All GI cancers	PreHTN → Normo	1427/40,859 (3.5)	326.8 (309.9–343.8)	1.00 (Reference)	-	1.00 (Reference)	-
	PreHTN → PreHTN	2405/62,600 (3.9)	355.7 (341.4–369.9)	1.09 (1.02–1.16)	0.012	1.01 (0.95–1.08)	0.705
	PreHTN → HTN	1378/27,708 (5.0)	466.1 (441.5–490.7)	1.42 (1.32–1.53)	<0.001	1.16 (1.08–1.26)	<0.001
Esophagus	PreHTN → Normo	28/40,859 (0.1)	6.5 (4.1–8.8)	1.00 (Reference)	-	1.00 (Reference)	-
	PreHTN → PreHTN	48/62,600 (0.1)	7.1 (5.1–9.2)	1.10 (0.69–1.75)	0.686	1.01 (0.63–1.62)	0.958
	PreHTN → HTN	38/27,708 (0.2)	12.9 (8.8–17.0)	1.99 (1.22–3.24)	0.006	1.63 (0.98–2.73)	0.063
Stomach	PreHTN → Normo	592/40,859 (1.5)	135.6 (124.7–146.5)	1.00 (Reference)	-	1.00 (Reference)	-
	PreHTN → PreHTN	925/62,600 (1.5)	136.8 (128.0–145.6)	1.01 (0.91–1.12)	0.873	0.94 (0.85–1.04)	0.231
	PreHTN → HTN	495/27,708 (1.8)	167.5 (152.7–182.2)	1.23 (1.09–1.39)	0.001	1.06 (0.93–1.20)	0.394
Colon and rectum	PreHTN → Normo	363/40,859 (0.9)	83.2 (74.6–91.7)	1.00 (Reference)	-	1.00 (Reference)	-
	PreHTN → PreHTN	703/62,600 (1.2)	104.0 (96.3–111.7)	1.25 (1.10–1.42)	<.001	1.14 (1.00–1.30)	0.044
	PreHTN → HTN	390/27,708 (1.5)	132.0 (118.9–145.1)	1.59 (1.37–1.83)	<.001	1.23 (1.06–1.43)	0.007
Liver	PreHTN → Normo	265/40,859 (0.7)	60.7 (53.4–68.0)	1.00 (Reference)	-	1.00 (Reference)	-
	PreHTN → PreHTN	409/62,600 (0.7)	60.5 (54.7–66.4)	1.00 (0.85–1.16)	0.964	0.97 (0.83–1.13)	0.654
	PreHTN → HTN	248/27,708 (0.9)	83.9 (73.5–94.4)	1.38 (1.16–1.64)	<0.001	1.18 (0.98–1.42)	0.076
Gallbladder and biliary tract	PreHTN → Normo	103/40,859 (0.3)	23.6 (19.1–28.2)	1.00 (Reference)	-	1.00 (Reference)	-
	PreHTN → PreHTN	142/62,600 (0.3)	21.0 (17.6–24.5)	0.89 (1.69–1.15)	0.366	0.80 (0.62–1.04)	0.093
	PreHTN → HTN	113/27,708 (0.5)	38.3 (31.2–45.3)	1.62 (1.24–2.12)	<0.001	1.10 (0.83–1.46)	0.523
Pancreas	PreHTN → Normo	76/40,859 (0.2)	17.5 (13.5–21.4)	1.00 (Reference)	-	1.00 (Reference)	-
	PreHTN → PreHTN	178/62,600 (0.3)	26.4 (22.5–30.2)	1.51 (1.15–1.97)	0.003	1.45 (1.11–1.91)	0.007
	PreHTN → HTN	94/27,708 (0.4)	31.8 (25.4–38.3)	1.81 (1.34–2.45)	<0.001	1.47 (1.07–2.02)	0.017

Abbreviations: BP, blood pressure; aHR, adjusted hazard ratio; HR, hazard ratio; CI, confidence interval; PY, person-years; HTN, hypertension; Normo, normotension; PreHTN, prehypertension.

**Table 3:** Subgroup analysis.

Variables	Subgroup	No. Events/Total (%)	aHR (95% CI)	*p* Value	*p* for Interaction
Age					0.004
≥65	PreHTN → Normo	320/4141 (7.8)	1.00 (Reference)	-	
	PreHTN → PreHTN	637/8168 (7.8)	0.98 (0.86–1.12)	0.765	
	PreHTN → HTN	581/7661 (7.6)	1.01 (0.88–1.17)	0.848	
<65	PreHTN → Normo	1107/36,718 (3.1)	1.00 (Reference)	-	
	PreHTN → PreHTN	1768/54,432 (3.3)	1.02 (0.94–1.10)	0.640	
	PreHTN → HTN	797/20,047 (4.0)	1.25 (1.13–1.37)	<0.001	
Sex					0.078
Men	PreHTN → Normo	925/20,038 (4.7)	1.00 (Reference)	-	
	PreHTN → PreHTN	1757/34,891 (5.1)	1.07 (0.98–1.16)	0.118	
	PreHTN → HTN	919/13,835 (6.7)	1.21 (1.10–1.33)	<0.001	
Women	PreHTN → Normo	502/20,821 (2.5)	1.00 (Reference)	-	
	PreHTN → PreHTN	648/27,709 (2.4)	0.91 (0.81–1.02)	0.117	
	PreHTN → HTN	459/13,873 (3.4)	1.08 (0.94–1.24)	0.277	
Diabetes					0.044
Yes	PreHTN → Normo	303/6293 (4.8)	1.00 (Reference)	-	
	PreHTN → PreHTN	540/10,547 (5.1)	1.02 (0.89–1.18)	0.790	
	PreHTN → HTN	461/8106 (5.7)	1.09 (0.94–1.27)	0.262	
No	PreHTN → Normo	1124/34,566 (3.3)	1.00 (Reference)	-	
	PreHTN → PreHTN	1865/52,053 (3.6)	1.01 (0.94–1.09)	0.824	
	PreHTN → HTN	917/19,602 (4.7)	1.19 (1.09–1.31)	<.001	
Smoking status					0.086
Never	PreHTN → Normo	918/29,815 (3.1)	1.00 (Reference)	-	
	PreHTN → PreHTN	1439/44,535 (3.2)	0.94 (0.87–1.03)	0.178	
	PreHTN → HTN	947/21,124 (4.5)	1.13 (1.03–1.24)	0.014	
Former	PreHTN → Normo	144/3754 (3.8)	1.00 (Reference)	-	
	PreHTN → PreHTN	298/6383 (4.7)	1.16 (0.94–1.41)	0.161	
	PreHTN → HTN	157/2462 (6.4)	1.35 (1.06–1.72)	0.014	
Current	PreHTN → Normo	365/7290 (5.0)	1.00 (Reference)	-	
	PreHTN → PreHTN	668/11,682 (5.7)	1.13 (0.99–1.29)	0.063	
	PreHTN → HTN	274/4122 (6.7)	1.15 (0.98–1.36)	0.086	

Abbreviations: aHR, adjusted hazard ratio; CI, confidence interval; HTN, hypertension; Normo, normotension; PreHTN, prehypertension.

**Table 4:** Site-specific GI cancer incidence and hazard ratios by BP trajectory and medication adherence.

Cancer Site	BP Trajectory from Prehypertension	Cases/Participants, *n* (%)	Incidence Rate per 100,000 PY	HR (95% CI)	*p* Value	aHR (95% CI)	*p* Value
All GI cancers	PreHTN → Normo	1427/40,859 (3.5)	326.8 (309.9–343.8)	1.00 (Reference)	-	1.00 (Reference)	-
	PreHTN → PreHTN	2405/62,600 (3.9)	355.7 (341.4–369.9)	1.09 (1.02–1.16)	0.012	1.01 (0.95–1.08)	0.705
	PreHTN → HTN	1378/27,708 (5.0)					
	with adherence (MPR) ^†^						
	MPR < 0.80	416/7423 (5.7)	536.9 (485.3–588.5)	1.64 (1.47–1.83)	<0.001	1.30 (1.16–1.45)	<0.001
	MPR ≥ 0.80	290/5969 (4.9)	472.5 (418.1–526.9)	1.45 (1.28–1.64)	<0.001	1.08 (0.95–1.23)	0.260
	Non-prescribed	672/14,316 (4.7)	428.7 (396.3–461.1)	1.31 (1.19–1.43)	<0.001	1.13 (1.03–1.24)	0.011
Esophagus	PreHTN → Normo	28/40,859 (0.1)	6.5 (4.1–8.8)	1.00 (Reference)	-	1.00 (Reference)	-
	PreHTN → PreHTN	48/62,600 (0.1)	7.1 (5.1–9.2)	1.10 (0.69–1.75)	0.686	1.01 (0.63–1.62)	0.958
	PreHTN → HTN	38/27,708 (0.2)					
	with adherence (MPR) ^†^						
	MPR < 0.80	13/7423 (0.2)	16.8 (7.7–25.9)	2.60 (1.35–5.03)	0.004	2.24 (1.12–4.47)	0.022
	MPR ≥ 0.80	10/5969 (0.2)	16.3 (6.2–26.4)	2.55 (1.24–5.25)	0.011	2.32 (1.07–5.03)	0.034
	Non-prescribed	15/14,316 (0.2)	9.6 (4.8–14.5)	1.47 (0.78–2.75)	0.231	1.19 (0.63–2.26)	0.595
Stomach	PreHTN → Normo	592/40,859 (1.5)	135.6 (124.7–146.5)	1.00 (Reference)	-	1.00 (Reference)	-
	PreHTN → PreHTN	925/62,600 (1.5)	136.8 (128.0–145.6)	1.01 (0.91–1.12)	0.873	0.94 (0.85–1.04)	0.231
	PreHTN → HTN	495/27,708 (1.8)					
	with adherence (MPR) ^†^						
	MPR < 0.80	143/7423 (2.0)	184.6 (154.3–214.8)	1.36 (1.13–1.63)	0.001	1.14 (0.94–1.38)	0.172
	MPR ≥ 0.80	102/5969 (1.8)	166.2 (134.0–198.5)	1.23 (0.99–1.51)	0.059	1.03 (0.82–1.28)	0.811
	Non-prescribed	250/14,316 (1.8)	159.5 (139.7–179.3)	1.17 (1.01–1.36)	0.034	1.03 (0.88–1.20)	0.726
Colon and rectum	PreHTN → Normo	363/40,859 (0.9)	83.2 (74.6–91.7)	1.00 (Reference)	-	1.00 (Reference)	-
	PreHTN → PreHTN	703/62,600 (1.2)	104.0 (96.3–111.7)	1.25 (1.10–1.42)	<0.001	1.14 (1.00–1.30)	0.044
	PreHTN → HTN	390/27,708 (1.5)					
	with adherence (MPR) ^†^						
	MPR < 0.80	113/7423 (1.6)	145.9 (119.0–172.8)	1.76 (1.42–2.17)	<0.001	1.30 (1.05–1.62)	0.019
	MPR ≥ 0.80	75/5969 (1.3)	122.2 (94.6–149.9)	1.47 (1.15–1.89)	0.002	1.06 (0.82–1.37)	0.675
	Non-prescribed	202/14,316 (1.5)	128.9 (111.1–146.7)	1.55 (1.30–1.84)	<0.001	1.26 (1.06–1.50)	0.011
Liver	PreHTN → Normo	265/40,859 (0.7)	60.7 (53.4–68.0)	1.00 (Reference)	-	1.00 (Reference)	-
	PreHTN → PreHTN	409/62,600 (0.7)	60.5 (54.7–66.4)	1.00 (0.85–1.16)	0.964	0.97 (0.83–1.13)	0.654
	PreHTN → HTN	248/27,708 (0.9)					
	with adherence (MPR) ^†^						
	MPR < 0.80	85/7423 (1.2)	109.7 (86.4–133.1)	1.81 (1.42–2.31)	<0.001	1.52 (1.18–1.95)	0.001
	MPR ≥ 0.80	49/5969 (0.9)	79.9 (57.5–102.2)	1.32 (0.97–1.79)	0.075	0.91 (0.66–1.26)	0.576
	Non-prescribed	114/14,316 (0.8)	72.8 (59.4–86.1)	1.20 (0.96–1.49)	0.110	1.13 (0.90–1.42)	0.281
Gallbladder and biliary tract	PreHTN → Normo	103/40,859 (0.3)	23.6 (19.1–28.2)	1.00 (Reference)	-	1.00 (Reference)	-
	PreHTN → PreHTN	142/62,600 (0.3)	21.0 (17.6–24.5)	0.89 (1.69–1.15)	0.366	0.80 (0.62–1.04)	0.093
	PreHTN → HTN	113/27,708 (0.5)					
	with adherence (MPR) ^†^						
	MPR < 0.80	31/7423 (0.5)	40.1 (26.0–54.1)	1.70 (1.14–2.54)	0.010	1.05 (0.69–1.59)	0.838
	MPR ≥ 0.80	30/5969 (0.6)	48.9 (31.4–66.4)	2.07 (1.38–3.11)	<0.001	1.14 (0.74–1.75)	0.569
	Non-prescribed	52/14,316 (0.4)	33.2 (24.2–42.2)	1.41 (1.01–1.96)	0.046	1.11 (0.79–1.56)	0.552
Pancreas	PreHTN → Normo	76/40,859 (0.2)	17.5 (13.5–21.4)	1.00 (Reference)	-	1.00 (Reference)	-
	PreHTN → PreHTN	178/62,600 (0.3)	26.4 (22.5–30.2)	1.51 (1.15–1.97)	0.003	1.45 (1.11–1.91)	0.007
	PreHTN → HTN	94/27,708 (0.4)					
	with adherence (MPR) ^†^						
	MPR < 0.80	31/7423 (0.5)	40.1 (26.0–54.1)	2.28 (1.50–3.46)	<0.001	1.75 (1.14–2.71)	0.011
	MPR ≥ 0.80	24/5969 (0.5)	39.2 (23.5–54.8)	2.24 (1.42–3.55)	0.001	1.67 (1.03–2.71)	0.039
	Non-prescribed	39/14,316 (0.3)	24.9 (17.1–32.7)	1.41 (0.96–2.08)	0.079	1.26 (0.85–1.86)	0.258

Abbreviations: BP, blood pressure; aHR, adjusted hazard ratio; HR, hazard ratio; CI, confidence interval; PY, person-years; HTN, hypertension; Normo, normotension; PreHTN, prehypertension; MPR, medication possession ratio. ^†^ Adherence (MPR): PreHTN → HTN progressor group subdivided by adherence, with PreHTN → Normo (reversion to normotension) as the reference.

## Data Availability

Restrictions apply to the availability of these data. The data were obtained from the Korean National Health Insurance Service (NHIS) claims database via the NHIS data platform (https://nhiss.nhis.or.kr/ (accessed on 6 February 2026)) and are not publicly available due to NHIS regulations and data access restrictions. The data may be accessed through the NHIS data access procedure upon reasonable request and approval. Further information is available from the corresponding author upon request.

## Supplementary Materials

The following supporting information can be downloaded at: https://www.mdpi.com/article/10.3390/cancers18040594/s1, Table S1: Results of landmark analyses by different exposure-window intervals; Table S2: Results of sensitivity analyses using varying lag time; Table S3: Results of sensitivity analyses using alternative BP classifications; Table S4: Baseline distribution of three BP classification by age group; Table S5: Results of subgroup analysis; Table S6: Baseline characteristics by BP trajectory from prehypertension with MPR subcategories; Figure S1: Timeline of study design; Figure S2: Flow chart of study population; Figure S3: Results of Schoenfeld residual test.

## Abbreviations

aHR	Adjusted Hazard Ratio
BMI	Body Mass Index
BP	Blood Pressure
CCI	Charlson Comorbidity Index
CI	Confidence Interval
GI	Gastrointestinal
ICD-10	International Classification of Diseases, 10th Revision
MPR	Medication Possession Ratio
NHIS	National Health Insurance Service
NHIS-HEALS	National Health Insurance Service–Health Screening Cohort
