# Supplementary material for: Progression from Prehypertension to Hypertension and Risk of Gastrointestinal Cancer—A Nationwide Health-Screening Cohort Study
Source: Cancers (Basel). 2026 Feb 11;18(4):594. doi: 10.3390/cancers18040594 (PMC12939411; doi:10.3390/cancers18040594)
Supplement: Supplementary file 1 [file cancers-18-00594-s001.zip › cancers-4088482-supplementary.pdf]

Supplementary Material

Progression from prehypertension to hypertension and risk of gastrointestinal cancer

: a nationwide health-screening cohort study

Supplementary Tables – pages 2 -9

Table S1: Results of landmark analyses by different exposure-window intervals – page 2

Table S2: Results of sensitivity analyses using varying lag time – page 3

Table S3: Results of sensitivity analyses using alternative BP classifications – page 4

Table S4: Baseline distribution of three BP classification by age group – page 5

Table S5: Results of subgroup analysis – page 6-7

Table S6: Baseline characteristics by BP trajectory from prehypertension with MPR subcategories – page 8-9

Supplementary Figures – pages 10 - 13

Supplementary Figure Legends – page 10

Figure S1: Timeline of study design – page 11

Figure S2: Flow chart of study population – page 12

Figure S3: Results of Schoenfeld residual test – page 13

Table S1. Results of landmark analyses by different exposure-window intervals

BP trajectory from prehypertension	Exposure window intervals		
	2 years	4 years	6 years
	aHR (95% CI)	aHR (95% CI)	aHR (95% CI)
PreHTN→Normo	1.00 (Reference)	1.00 (Reference)	1.00 (Reference)
PreHTN→PreHTN	1.05 (0.97-1.14)	1.01 (0.95–1.08)	1.02 (0.96-1.09)
PreHTN→HTN	1.26 (1.14-1.39)	1.16 (1.08–1.26)	1.15 (1.07-1.25)

Notes: Exposure windows of 2, 4, and 6 years between baseline and follow-up screenings were used to define blood pressure trajectory. The 4-year interval was used in the main analysis, and 2- and 6-year intervals were additionally tested to examine temporal robustness.

Abbreviations: BP, blood pressure; GI, gastrointestinal; HR, hazard ratio; aHR, adjusted hazard ratio; CI, confidence interval; PY, person-years; HTN, hypertension; Normo, normotension; PreHTN, prehypertension

Table S2. Results of sensitivity analyses using varying lag times

BP trajectory from prehypertension	Lag time				
	1 year	2 years	3 years	4 years	5 years
	aHR (95% CI)	aHR (95% CI)	aHR (95% CI)	aHR (95% CI)	aHR (95% CI)
PreHTN→Normo	1.00 (Reference)	1.00 (Reference)	1.00 (Reference)	1.00 (Reference)	1.00 (Reference)
PreHTN→PreHTN	1.02 (0.95-1.09)	1.01 (0.94-1.08)	1.00 (0.93-1.08)	0.97 (0.90-1.06)	0.96 (0.88-1.05)
PreHTN→HTN	1.16 (1.07-1.26)	1.16 (1.06-1.26)	1.17 (1.07-1.28)	1.14 (1.03-1.25)	1.13 (1.02-1.25)

Notes: Lag times were defined as 1 to 5 years after the index date. Only participants alive and cancer-free at each lag time point were included.

Abbreviations: BP, blood pressure; GI, gastrointestinal; HR, hazard ratio; aHR, adjusted hazard ratio; CI, confidence interval; PY, person-years; HTN, hypertension; Normo, normotension; PreHTN, prehypertension

Table S3. Results of sensitivity analyses using alternative BP classifications

BP classification	BP trajectory from prehypertension	Cases/participants, n (%)	Incidence rate per 100,000 PY	HR (95% CI)	P value	aHR (95% CI)	P value
JNC 7	PreHTN→Normo (SBP<120 and DBP<80)	1,427/40,859 (3.5)	326.8 (309.9–343.8)	1.00 (Reference)	-	1.00 (Reference)	
	PreHTN→PreHTN (SBP 120-139 or DBP 80-90)	2,405/62,600 (3.9)	355.7 (341.4–369.9)	1.09 (1.02-1.16)	0.012	1.01 (0.95–1.08)	0.71
	PreHTN→HTN (SBP≥140 or DBP≥90)	1,378/27,708 (5.0)	466.1 (441.5–490.7)	1.42 (1.32-1.53)	<0.001	1.16 (1.08–1.26)	<0.001
ESC 2024	PreHTN→Normo (SBP<120 and DBP<70)	777/23,820 (3.3)	622.1 (578.4-665.9)	1.00 (Reference)	-	1.00 (Reference)	-
	PreHTN→PreHTN (SBP 120-139 or DBP 70-89)	4,014/109,425 (3.7)	334.9 (324.5-345.2)	1.11 (1.02-1.19)	0.010	0.99 (0.92-1.07)	0.77
	PreHTN→HTN (SBP≥140 or DBP≥90)	1,537/30,566 (5.1)	467.9 (444.5-491.3)	1.55 (1.42-1.68)	<.001	1.16 (1.06-1.27)	0.001
ACC/AHA 2017	PreHTN→Normo (SBP<120 and DBP<80)	414/10,757 (3.9)	364.2 (329.2-399.3)	1.00 (Reference)	-	1.00 (Reference)	-
	PreHTN→PreHTN (SBP 120-129 and DBP<80)	161/4,205 (3.8)	358.3 (303.0-413.7)	0.98 (0.82-1.18)	0.86	0.98 (0.82-1.18)	0.86
	PreHTN→HTN Stage 1 (SBP 130-139 or DBP 80-89)	368/9,078 (4.1)	378.9 (340.2-417.6)	1.04 (0.90-1.20)	0.58	1.00 (0.87-1.16)	0.95
	PreHTN→HTN Stage 2 (SBP≥140 or DBP≥90)	155/3,011 (5.2)	486.9 (410.2-563.5)	1.34 (1.11-1.61)	0.002	1.28 (1.05-1.54)	0.013

Notes: In the main analysis, PreHTN was defined according to the JNC7 criteria (SBP 120–139 mmHg or DBP 80–89 mmHg). For the sensitivity analyses, "elevated BP" defined by the 2024 ESC classification (SBP 120–139 mmHg or DBP 70–89 mmHg) and by the 2017 ACC/AHA classification (SBP 120–129 mmHg and DBP < 80 mmHg) was also categorized as PreHTN.

Abbreviations: BP, blood pressure; SBP, systolic blood pressure; DBP, diastolic blood pressure; HR, hazard ratio; aHR, adjusted hazard ratio; CI, confidence interval; PY, person-years; HTN, hypertension; Normo, normotension; PreHTN, prehypertension; JNC 7, The seventh report of the Joint National Committee; ESC, European Society of Cardiology; ACC/AHA, American College of Cardiology/American Heart Association

Table S4. Baseline distribution of three BP classification by age group

BP classification	Category	Age group (n)					Total
		40-49	50-59	60-69	70-79	≥80	
JNC 7	Normal	14,152	17,450	6,774	2,282	201	40,859
	Prehypertension	19,883	25,749	12,034	4,532	402	62,600
	Hypertension	5,099	9,534	7,995	4,565	515	27,708
	Total	39,134	52,733	26,803	11,379	1,118	131,167
ESC 2024	Non-elevated (Normal)	8,594	10,128	3,786	1,204	108	23,820
	Elevated	37,445	44,755	19,725	6,898	602	109,425
	Hypertension	5,682	10,421	8,855	5,047	561	30,566
	Total	51,721	65,304	32,366	13,149	1,271	163,811
ACC/AHA 2017	Normal	3,380	4,440	2,064	799	74	10,757
	Elevated	1,116	1,659	937	456	37	4,205
	Stage 1	2,356	3,430	2,104	1,083	105	9,078
	Stage 2	440	898	950	640	83	3,011
	Total	7,292	10,427	6,055	2,978	299	27,051

Notes: Counts are numbers of participants at baseline screening (t0). Age groups are in years. Categories reflect each guideline's office BP thresholds. Totals may differ across panels because category definitions vary by classification.

Abbreviations: BP, blood pressure; SBP, systolic blood pressure; DBP, diastolic blood pressure; HR, hazard ratio; aHR, adjusted hazard ratio; CI, confidence interval; PY, person-years; HTN, hypertension; Normo, normotension; PreHTN, prehypertension; JNC 7, The seventh report of the Joint National Committee; ESC, European Society of Cardiology; ACC/AHA, American College of Cardiology/American Heart Association

Table S5. Results of subgroup analysis

Variables	Subgroup	No. events/total (%)	aHR (95% CI)	P value	P for interaction
**Age**					0.004
≥65	PreHTN→Normo	320/4,141 (7.8)	1.00 (Reference)	-	
	PreHTN→PreHTN	637/8,168 (7.8)	0.98 (0.86-1.12)	0.77	
	PreHTN→HTN	581/7,661 (7.6)	1.01 (0.88-1.17)	0.85	
<65	PreHTN→Normo	1,107/36,718 (3.1)	1.00 (Reference)	-	
	PreHTN→PreHTN	1,768/54,432 (3.3)	1.02 (0.94-1.10)	0.64	
	PreHTN→HTN	797/20,047 (4.0)	1.25 (1.13-1.37)	<0.001	
**Sex**					0.078
Men	PreHTN→Normo	925/20,038 (4.7)	1.00 (Reference)	-	
	PreHTN→PreHTN	1,757/34,891 (5.1)	1.07 (0.98-1.16)	0.12	
	PreHTN→HTN	919/13,835 (6.7)	1.21 (1.10-1.33)	<0.001	
Women	PreHTN→Normo	502/20,821 (2.5)	1.00 (Reference)	-	
	PreHTN→PreHTN	648/27,709 (2.4)	0.91 (0.81-1.02)	0.12	
	PreHTN→HTN	459/13,873 (3.4)	1.08 (0.94-1.24)	0.28	
**BMI (kg/m^2)**					0.47
Normal or underweight	PreHTN→Normo	681/20,209 (3.4)	1.00 (Reference)	-	
	PreHTN→PreHTN	948/24,209 (3.9)	1.06 (0.96-1.17)	0.22	
	PreHTN→HTN	464/8,666 (5.4)	1.25 (1.10-1.41)	<0.001	
Overweight	PreHTN→Normo	404/11,431 (3.5)	1.00 (Reference)	-	
	PreHTN→PreHTN	691/18,636 (3.7)	0.96 (0.85-1.09)	0.56	
	PreHTN→HTN	379/8,058 (4.7)	1.07 (0.92-1.24)	0.38	
Obese	PreHTN→Normo	342/9,219 (3.7)	1.00 (Reference)	-	
	PreHTN→PreHTN	766/19,755 (3.9)	0.98 (0.86-1.11)	0.73	
	PreHTN→HTN	535/10,984 (4.9)	1.13 (0.98-1.31)	0.084	
**Drinking habits**					0.55
Rarely	PreHTN→Normo	1,061/32,065 (3.3)	1.00 (Reference)	-	
	PreHTN→PreHTN	1,718/47,345 (3.6)	1.01 (0.94-1.09)	0.80	
	PreHTN→HTN	995/21,779 (4.6)	1.12 (1.02-1.23)	0.013	
1~4 times per week	PreHTN→Normo	289/7,740 (3.7)	1.00 (Reference)	-	
	PreHTN→PreHTN	526/13,046 (4.0)	1.02 (0.88-1.18)	0.76	
	PreHTN→HTN	242/4,640 (5.2)	1.21 (1.01-1.45)	0.035	
5~7 times per week	PreHTN→Normo	77/1,054 (7.3)	1.00 (Reference)	-	
	PreHTN→PreHTN	161/2,209 (7.3)	1.00 (0.76-1.31)	0.98	
	PreHTN→HTN	141/1,289 (10.9)	1.41 (1.06-1.88)	0.020	
**Physical activity**					0.21
Rarely	PreHTN→Normo	674/17,897 (3.8)	1.00 (Reference)	-	
	PreHTN→PreHTN	1,131/28,111 (4.0)	0.99 (0.89-1.08)	0.76	
	PreHTN→HTN	756/14,081 (5.4)	1.16 (1.04-1.29)	0.009	
1~4 times per week	PreHTN→Normo	561/17,811 (3.2)	1.00 (Reference)	-	
	PreHTN→PreHTN	967/26,923 (3.6)	1.06 (0.95-1.18)	0.28	
	PreHTN→HTN	463/10,162 (4.6)	1.22 (1.07-1.39)	0.003	
5~7 times per week	PreHTN→Normo	192/5,151 (3.7)	1.00 (Reference)	-	
	PreHTN→PreHTN	307/7,566 (4.1)	0.98 (0.82-1.17)	0.81	
	PreHTN→HTN	159/3,465 (4.6)	1.02 (0.82-1.27)	0.88	
**Smoking status**					0.086
Never	PreHTN→Normo	918/29,815 (3.1)	1.00 (Reference)	-	
	PreHTN→PreHTN	1,439/44,535 (3.2)	0.94 (0.87-1.03)	0.18	
	PreHTN→HTN	947/21,124 (4.5)	1.13 (1.03-1.24)	0.014	
Former	PreHTN→Normo	144/3,754 (3.8)	1.00 (Reference)	-	

Variables	Subgroup	No. events/total (%)	aHR (95% CI)	P value	P for interaction
	PreHTN→PreHTN	298/6,383 (4.7)	1.16 (0.94-1.41)	0.16	
	PreHTN→HTN	157/2,462 (6.4)	1.35 (1.06-1.72)	0.014	
Current	PreHTN→Normo	365/7,290 (5.0)	1.00 (Reference)	-	
	PreHTN→PreHTN	668/11,682 (5.7)	1.13 (0.99-1.29)	0.063	
	PreHTN→HTN	274/4,122 (6.7)	1.15 (0.98-1.36)	0.086	
**Diabetes**					0.044
Yes	PreHTN→Normo	303/6,293 (4.8)	1.00 (Reference)	-	
	PreHTN→PreHTN	540/10,547 (5.1)	1.02 (0.89-1.18)	0.79	
	PreHTN→HTN	461/8,106 (5.7)	1.09 (0.94-1.27)	0.26	
No	PreHTN→Normo	1,124/34,566 (3.3)	1.00 (Reference)	-	
	PreHTN→PreHTN	1,865/52,053 (3.6)	1.01 (0.94-1.09)	0.82	
	PreHTN→HTN	917/19,602 (4.7)	1.19 (1.09-1.31)	<.001	
**Dyslipidemia**					0.55
Yes	PreHTN→Normo	325/9,074 (3.6)	1.00 (Reference)	-	
	PreHTN→PreHTN	549/14,518 (3.8)	0.97 (0.85-1.12)	0.68	
	PreHTN→HTN	488/10,218 (4.8)	1.13 (0.98-1.31)	0.094	
No	PreHTN→Normo	1,102/31,785 (3.5)	1.00 (Reference)	-	
	PreHTN→PreHTN	1,856/48,082 (3.9)	1.02 (0.95-1.11)	0.53	
	PreHTN→HTN	890/17,490 (5.1)	1.17 (1.07-1.29)	0.001	
**Insulin or glucose–lowering agents**					0.66
Yes	PreHTN→Normo	107/2.045 (5.2)	1.00 (Reference)	-	
	PreHTN→PreHTN	225/3,780 (6.0)	1.14 (0.91-1.44)	0.26	
	PreHTN→HTN	232/3,406 (6.8)	1.32 (1.04-1.67)	0.023	
No	PreHTN→Normo	1.320/38,814 (3.4)	1.00 (Reference)	-	
	PreHTN→PreHTN	2,180/58,820 (3.7)	1.00 (0.93-1.07)	0.99	
	PreHTN→HTN	1,146/24,302 (4.7)	1.15 (1.05-1.25)	0.002	
**Lipid–lowering agents**					0.24
Yes	PreHTN→Normo	186/6,124 (3.0)	1.00 (Reference)	-	
	PreHTN→PreHTN	375/9,828 (3.8)	1.19 (1.00-1.42)	0.058	
	PreHTN→HTN	336/7,040 (4.8)	1.39 (1.15-1.68)	0.001	
No	PreHTN→Normo	1,241/34,735 (3.6)	1.00 (Reference)	-	
	PreHTN→PreHTN	2,030/52,772 (3.9)	0.99 (0.92-1.06)	0.70	
	PreHTN→HTN	1,042/20,668 (5.0)	1.12 (1.03-1.23)	0.008	

Abbreviations: aHR, adjusted hazard ratio; CI, confidence interval; PY, person-years; HTN, hypertension; Normo, normotension; PreHTN, prehypertension;

Table S6. Baseline characteristics by BP trajectory from prehypertension with MPR subcategories

Variables	BP trajectory from prehypertension						P value
	Overall	Reversion to normotension	Persistence of prehypertension	Progression to hypertension			
				MPR<0.8	MPR≥0.8	Non-prescribed	
**Participants, n**	131,167	40,859	62,600	7,423	5,969	14,316	
**Follow-up period (years) †**	10.7 (2.6)	10.7 (2.5)	10.8 (2.5)	10.4 (3.2)	10.3 (3.0)	11.0 (2.7)	<0.001
**Blood pressure**							
Systolic (mmHg) †	124.0 (7.0)	122.4 (6.9)	124.1 (6.9)	126.1 (6.9)	126.4 (6.8)	125.6 (7.0)	<0.001
Diastolic (mmHg) †	77.7 (5.6)	77.2 (5.7)	77.9 (5.5)	78.1 (5.8)	78.1 (5.7)	78.4 (5.5)	<0.001
**First screening year (years)**							<0.001
2003	71,793 (54.7)	19,469 (47.6)	34,442 (55.0)	4,880 (65.7)	3848 (64.5)	9154 (63.9)	
2004	31,161 (23.8)	10,532 (25.8)	15,072 (24.1)	1,428 (19.2)	1140 (19.1)	2989 (20.9)	
2005	12,498 (9.5)	4,829 (11.8)	5,795 (9.3)	475 (6.4)	450 (7.5)	949 (6.6)	
2006	15,715 (12.0)	6,029 (14.8)	7,291 (11.6)	640 (8.6)	531 (8.9)	1224 (8.5)	
**Age (years) †**	55.52 (8.70)	53.98 (7.85)	54.90 (8.37)	61.0 (9.5)	62.0 (9.2)	57.1 (9.2)	<0.001
**Aged 65 or above**	19,970 (15.2)	4,141 (10.1)	8,168 (13.0)	2,498 (33.7)	2,259 (37.8)	2,904 (20.3)	<0.001
**Women**	62,403 (47.6)	20,821 (51.0)	27,709 (44.3)	4,076 (54.9)	3,244 (54.3)	6,553 (45.8)	<0.001
**Region**							<0.001
Metropolitan	21,041 (16.0)	6,719 (16.4)	9,851 (15.7)	1,066 (14.4)	1,001 (16.8)	2,404 (16.8)	
Urban	38,195 (29.1)	12,198 (29.9)	18,907 (30.2)	1,817 (24.5)	1,586 (26.6)	3,687 (25.8)	
Rural	71,931 (54.8)	21,942 (53.7)	33,842 (54.1)	4,540 (61.2)	3,382 (56.7)	8,225 (57.5)	
**Household insurance rate**							
Low (≤5th decile)	45,058 (34.4)	13,620 (33.3)	21,027 (33.6)	2,897 (39.0)	2,178 (36.5)	5,336 (37.3)	
High (6–10th decile)	86,109 (65.6)	27,239 (66.7)	41,573 (66.4)	4,526 (61.0)	3,791 (63.5)	8,980 (62.7)	<0.001
**Disability**	7,867 (6.0)	2,100 (5.1)	3,552 (5.7)	715 (9.6)	571 (9.6)	929 (6.5)	<0.001
**Family history**							
**Hypertension**	36,605 (27.9)	10,103 (24.7)	17,057 (27.2)	2,626 (35.4)	2,525 (42.3)	4,294 (30.0)	<0.001
**Cancer**	47,424 (36.2)	15,868 (38.8)	22,671 (36.2)	2,295 (30.9)	1,838 (30.8)	4,752 (33.2)	<0.001

**CCI**							<0.001
0	96,293 (73.4)	29,551 (72.3)	46,991 (75.1)	5,043 (67.9)	3,361 (56.3)	11,347 (79.3)	
1	20,272 (15.5)	6,736 (16.5)	9,345 (14.9)	1,144 (15.4)	1,232 (20.6)	1,815 (12.7)	
2	8,819 (6.7)	2,823 (6.9)	3,917 (6.3)	644 (8.7)	718 (12.0)	717 (5.0)	
≥3	5,783 (4.4)	1,749 (4.3)	2,347 (3.7)	592 (8.0)	658 (11.0)	437 (3.1)	
**Diabetes**	24,946 (19.0)	6,293 (15.4)	10,547 (16.8)	2,695 (36.3)	2,625 (44.0)	2,786 (19.5)	<0.001
**Dyslipidemia**	33,810 (25.8)	9,074 (22.2)	14,518 (23.2)	3,249 (43.8)	3,490 (58.5)	2,479 (24.3)	<0.001
**Insulin or glucose–lowering agents**	9,231 (7.0)	2,045 (5.0)	3,780 (6.0)	1,133 (15.3)	1,250 (20.9)	1,023 (7.1)	<0.001
**Lipid–lowering agents**	22,992 (17.5)	6,124 (15)	9,828 (15.7)	2,294 (30.9)	2,408 (40.3)	2,338 (16.3)	<0.001
**BMI (kg/m^2)**							<0.001
Normal or underweight	53,084 (40.5)	20,209 (49.5)	24,209 (38.7)	2,331 (31.4)	1,663 (27.9)	4,672 (32.6)	
Overweight	38,125 (29.1)	11,431 (28.0)	18,636 (29.8)	2,081 (28.0)	1,772 (29.7)	4,205 (29.4)	
Obese	39,958 (30.5)	9,219 (22.6)	19,755 (31.6)	3,011 (40.6)	2,534 (42.5)	5,439 (38.0)	
**Total cholesterol (mg/dL) †**	199.3 (36.5)	196.3 (35.5)	200.1 (36.1)	203.2 (39.5)	197.3 (39.5)	203.1 (37.3)	<0.001
**Alcohol consumption**							<0.001
Rarely	101,189 (77.1)	32,065 (78.5)	47,345 (75.6)	6,054 (81.6)	4,932 (82.6)	10,793 (75.4)	
1–4 times per week	25,426 (19.4)	7,740 (18.9)	13,046 (20.8)	1,046 (14.1)	826 (13.8)	2,768 (19.3)	
5–7 times per week	4,552 (3.5)	1,054 (2.6)	2,209 (3.5)	323 (4.4)	211 (3.5)	755 (5.3)	
**Physical activity**							<0.001
Rarely	60,089 (45.8)	17,897 (43.8)	28,111 (44.9)	4,053 (54.6)	2,963 (49.6)	7,065 (49.4)	
1–4 times per week	54,896 (41.9)	17,811 (43.6)	26,923 (43.0)	2,468 (33.2)	2,121 (35.5)	5,573 (38.9)	
5–7 times per week	16,182 (12.3)	5,151 (12.6)	7,566 (12.1)	902 (12.2)	885 (14.8)	1,678 (11.7)	
**Smoking status**							<0.001
Never	95,474 (72.8)	29,815 (73.0)	44,535 (71.1)	5,791 (78.0)	4,767 (79.9)	10,566 (73.8)	
Former	12,599 (9.6)	3,754 (9.2)	6,383 (10.2)	591 (8.0)	547 (9.2)	1,324 (9.2)	
Current	23,094 (17.6)	7,290 (17.8)	11,682 (18.7)	1,041 (14.0)	655 (11.0)	2,426 (16.9)	

† Mean and standard deviation

Abbreviations: CCI, Charlson Comorbidity Index; BMI, body mass index; MPR, medication possession ratio

SUPPLEMENTARY FIGURE LEGENDS

Figure S1: Study design and timeline showing the period for blood pressure trajectory assessment, index date setting, and follow-up for outcome ascertainment.

Figure S2: Flow diagram showing the selection of the final study population from individuals with prehypertension after applying exclusion criteria.

Figure S3: Schoenfeld residuals were plotted to assess the proportional hazards assumption in the cause-specific Cox proportional hazards model.

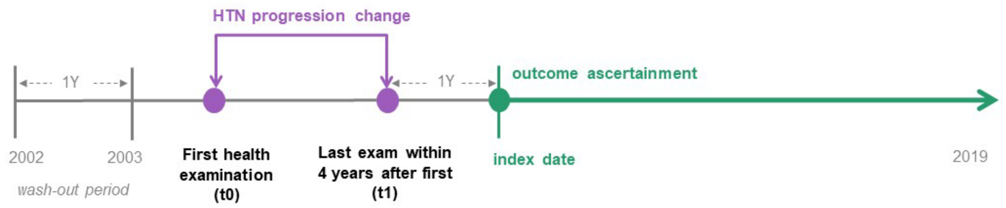

Figure S1. Timeline of study design.

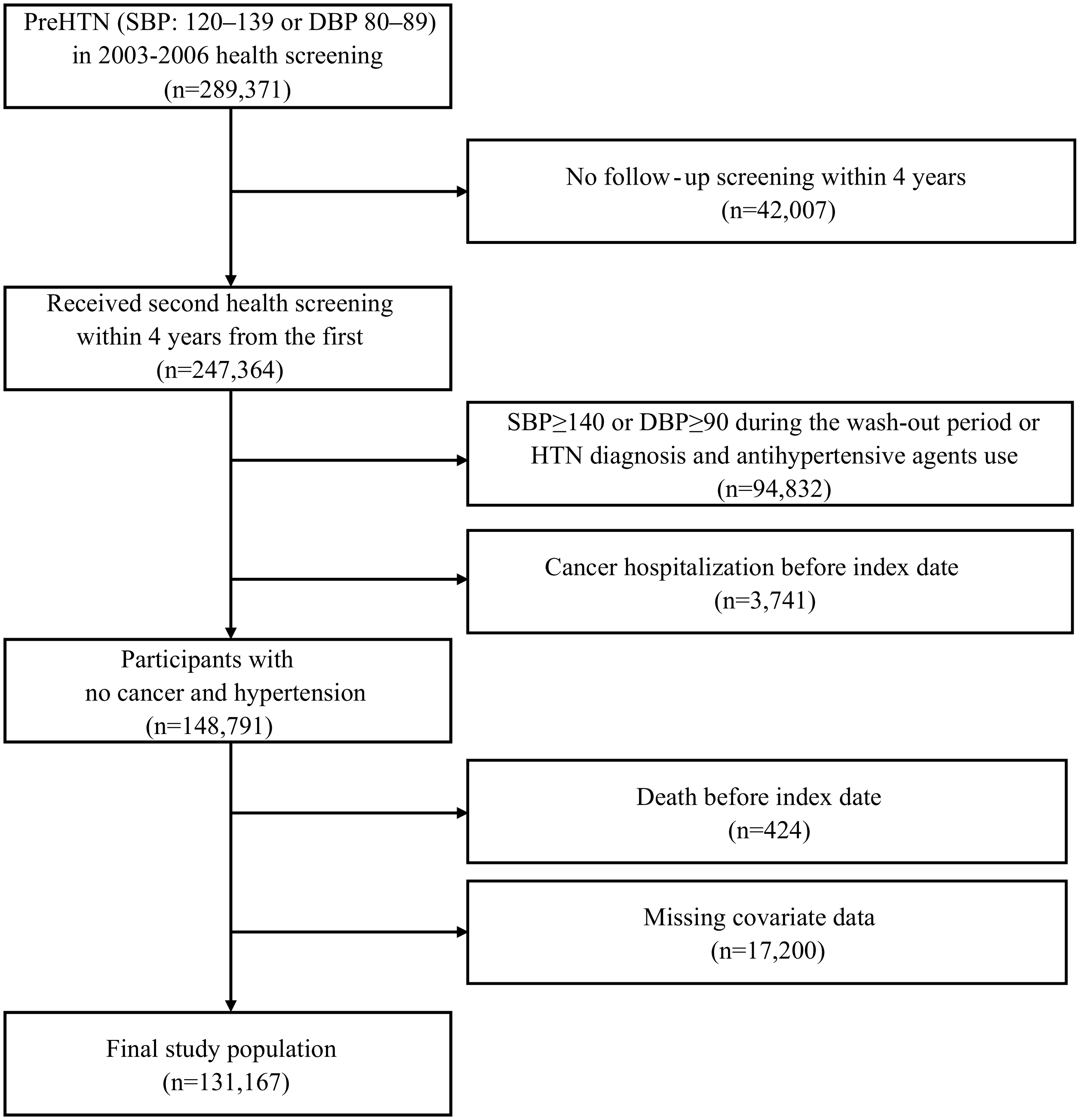

Figure S2. Flow chart of study population

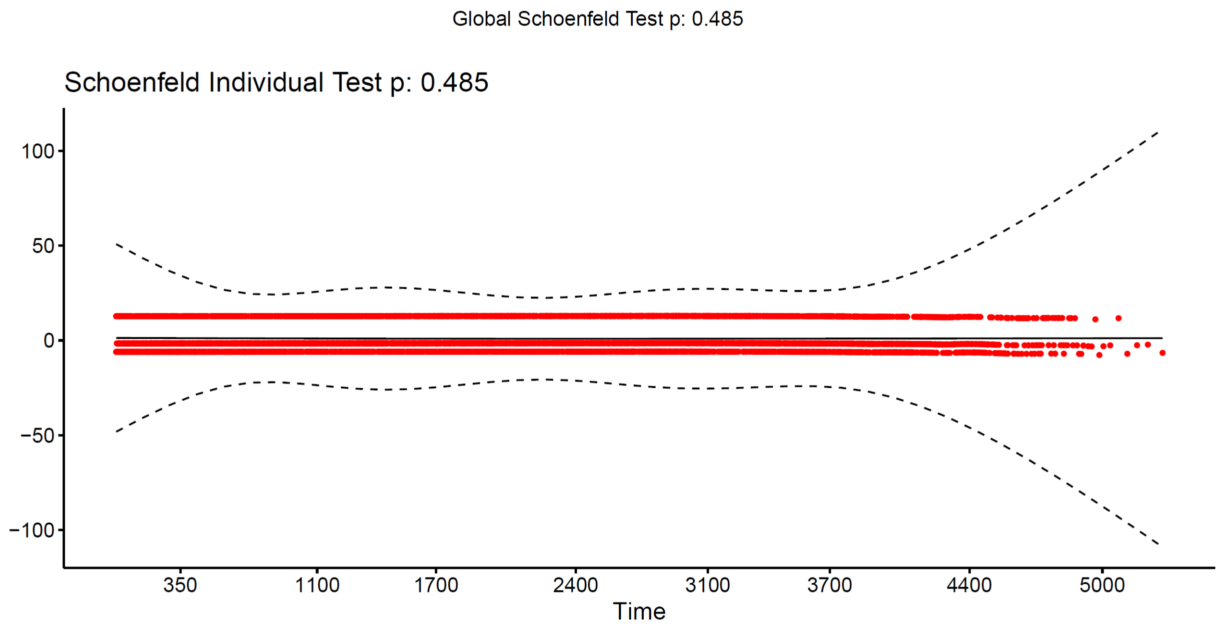

Figure S3. Results of Schoenfeld residual test
